# Efficacy of Laparoscopic Surgery Combined With Leuprorelin in the Treatment of Endometriosis Associated With Infertility and Analysis of Influencing Factors for Recurrence

**DOI:** 10.3389/fsurg.2022.873698

**Published:** 2022-04-19

**Authors:** Lu Yu, Yunming Sun, Qiongyan Fang

**Affiliations:** ^1^Department of Pharmacy, Zhoushan Women and Children Hospital, Zhoushan, China; ^2^Department of Gynaecology, Zhoushan Women and Children Hospital, Zhoushan, China; ^3^Department of Pharmacy, Zhoushan Hospital of Zhejiang Province, Zhoushan, China

**Keywords:** endometriosis, leuprorelin, laparoscopic surgery, efficacy, recurrence

## Abstract

**Objective:**

To explore the curative effect of laparoscopic surgery combined with leuprorelin in the treatment of endometriosis with infertility and the related factors of recurrence after treatment.

**Methods:**

A total of 158 patients with endometriosis and infertility were selected in our hospital from January 2019 to June 2020. Patients were randomly divided into the control group and the observation group, with 79 patients in each group. Patients in the control group was treated by laparoscopy surgery combined with dydrogesterone, while those in the observation group was treated with laparoscopic surgery combined with leuprorelin. The hormone levels, recurrence rate, pregnancy rate and adverse reaction of the two groups were compared. Combined with clinical and pathological information, the related factors of postoperative recurrence were analyzed.

**Results:**

After treatment, the levels of luteinizing hormone, follicle-stimulating hormone and estradiol in the observation group were lower than those in the control group (*P* < 0.05). The recurrence rate at 12 months after operation in the observation group was lower than that in the control group, and the pregnancy rate was higher than that in the control group (*P* < 0.05). However, there was no significant difference in the incidence of adverse reactions between the two groups (*P* > 0.05). Preoperative dysmenorrhea was an independent risk factor for postoperative recurrence in patients with endometriosis, and postoperative pregnancy was a protective factor for postoperative recurrence in patients with endometriosis (*P* < 0.05).

**Conclusion:**

Laparoscopy combined with leuprorelin in the treatment of endometriosis with infertility can improve hormone levels, increase the pregnancy rate and reduce the recurrence rate. Preoperative dysmenorrhea is an independent risk factor for postoperative recurrence, which should be quantified and included in the recurrence risk prediction model. Postoperative pregnancy can reduce the recurrence rate after operation, and patients with fertility requirements should be encouraged to make activ preparations for postoperative pregnancy.

## Introduction

Endometriosis is a common gynecological disease, which is characterized by the fact that active endometrial cells are planted outside the endometrium. Uterus is connected to the pelvis through fallopian tubes. During the menstrual period, menstrual reflux, abnormal secretion of gonad hormones, abnormal stimulation of inflammatory reaction, abnormal metastasis of lymphatic system, defects of immune system, etc. Will make endometrial cells grow outside endometrium and cause endometriosis (1, 2). Among them, pelvic organs and parietal peritoneum are the most commonly involved parts of ectopic endometrium. Women of childbearing age are the main patients with endometriosis, and the clinical manifestations of endometriosis are dysmenorrhea, abnormal menstruation, and sexual pain (3). When the staging standard of endometriosis reaches the stage III-IV of the revised American Fertility Society classification (r-AFS) scoring system, it will lead to infertility and greatly affect the daily life and fertility of patients (4).

At present, laparoscopic surgery is often used in clinical treatment of endometriosis. Because of its advantages such as less trauma and rapid postoperative recovery, most patients need conservative surgery to preserve their ovary. However, the fertility of patients with endometriosis who have infertility symptoms has not improved significantly. In recent years, clinical observation and related data show that laparoscopic surgery alone in the treatment of moderate to severe endometriosis patients with low actual cure rate, high recurrence rate and low success rate of postoperative pregnancy (5, 6). Therefore, some scholars suggest that laparoscopic surgery combined with drug therapy can promote the recovery of patients' fertility (7). Leuprorelin is a gonadotropin which can inhibit the function of the pituitary-gonadal system, inhibit the secretion of estrogen, regulate the level of ovarian hormone and promote pregnancy. In this study, laparoscopic surgery combined with leuprorelin was used to treat endometriosis with infertility, to explore the effect of this method on the hormone level and pregnancy rate, and to analyze the clinical records and follow-up data, so as to further explore the risk factors for postoperative recurrence, and to provide a theoretical basis for the treatment and prevention of recurrence of endometriosis with infertility.

## Data and Methods

### General Information

A total of 158 patients with endometriosis associated with infertility were selected from January 2019 to June 2020 in our hospital and divided into the control group and the observation group using the random number method, with 79 patients in each group. Inclusion criteria: (1) Not pregnant for more than 1 year without contraception; (2) Ultrasonography showed that there was growth and infiltration outside endometrium, and repeated bleeding formed nodules and masses, which caused pain; (3) Patients who did not take hormone drugs within 6 months before the study; (4) The clinical data are complete. Exclusion criteria: (1) Cognitive dysfunction or mental illness; (2) Accompanied with dysfunction of heart, liver, kidney and other organs; (3) There are contraindications for operation; (4) There are drug contraindications; (5) Failed to meet the follow-up conditions. The control group: The age was (28.91 ± 3.06) years old, BMI was (22.19 ± 0.84) kg/m^2^, and the duration of infertility was (4.13 ± 0.54) years. The observation group: The age was (29.43 ± 3.17) years old, BMI was (22.37 ± 0.92) kg/m^2^ and the duration of infertility was (4.28 ± 0.62) years. There was no significant difference in general information such as age between the two groups (*P* > 0.05), indicating that they were comparable.

### Research Methods

#### Laparoscopic Surgery

Both groups underwent routine examination after admission, and the operation time was from day 3 to day 7 after menstrual period. After general anesthesia, CO_2_ pneumoperitoneum was performed. Various organs and tissues in the pelvic cavity and peritoneum were explored under the guidance of laparoscope, to master the specific location and scope of the lesions. According to the position, size and distribution of the lesions. For patients with pelvic adhesions, pelvic adhesions should be removed, and pelvic anatomy should be restored as quickly as possible. Electrocoagulation was performed in patients with obvious punctate deposits to reduce and remove ectopic focus. When clearing the ovarian cyst, the normal ovarian tissue should be kept as much as possible, and the ovary should be sutured after the operation to restore the normal morphology. During the operation, under the condition of not damaging bilateral ovaries and uterus, electrocoagulation was performed on the tiny lesions located on the surface of uterus, sacral ligament, peritoneum and pelvic wall. Then the pelvis was rinsed with normal saline, and sodium hyaluronate gel was applied to the wound surface to prevent secondary adhesion after the operation. After treatment, the patients in the two groups were followed up for 12 months.

#### The Control Group Was Treated With Laparoscopic Surgery Combined With Dydrogestone

On day 3 after laparoscopic surgery, 10 mg of dydrogestone (Manufacturer: Abbott Biologicals B.V, Registration CertificateNo. H20130110) was administered orally, twice a week, for a continuous period of 3 months.

#### Observation Group Using Laparoscopic Surgery Combined With Leuprorelin Treatment

The patient was given three subcutaneous injections of leuprorelin (Manufacturer: Ipsen Pharma Biotech, registration number H20090247) 3.75 mg on the 4th or 5th day after surgery, once every 1 menstrual cycle and for 3 months.

#### Observation Indicators

(1) Comparison of hormone levels between the two groups: On the 2nd and 3rd day of menstrual period before and after treatment, 4 ml of fasting venous blood were collected in the morning, and the hormone levels of luteinizing hormone, follicle stimulating hormone and estradiol were detected by ELISA after centrifugation.

(2) Comparison of the recurrence after 12 months between the two groups. Recurrence rate = number of recurrent cases/total cases ×100%.

(3) Comparison of pregnancy rates between the two groups: The number of successful pregnancies within 12 months after surgery in the two groups was counted. Successful pregnancy: On day 45 of gestation, gestational sac was found in the uterus by ultrasonography. Pregnancy rate = cases of successful pregnancy/total cases ×100%.

(4) Comparison of the incidence of adverse reactions between the two groups: The incidence of adverse reactions such as abnormal liver function, gastrointestinal reactions and irregular vaginal bleeding after treatment in the two groups was counted. Incidence of adverse reactions = cases of adverse reactions/total cases ×100%.

(5) According to the recurrence of patients after surgery, they were divided into recurrence group and non-recurrence group. The clinical medical records were collected to record the dysmenorrhea, r-AFS staging, unilateral/bilateral, maximum cyst diameter, rupture or not, and postoperative medication of the patients. Return visit was mainly conducted through telephone call and outpatient re-examination, to collect information about patients' postoperative recurrence and pregnancy. Criteria for recurrence: B-scan ultrasonography after surgery revealed new ovarian cysts, in which dense punctiform echoes with a diameter of at least 2 cm were more common, and the cysts did not disappear after several consecutive menstrual cycles, with or without elevated CA-125 and dysmenorrhea. To analyze the related factors of recurrence of endometriosis after treatment.

### Statistical Methods

SPSS22.0 software was used for processing. Experimental data the measurement data such as luteinizing hormone, follicle stimulating hormone and estradiol level are expressed by mean standard deviation (*x*±s), and the counting data such as recurrence rate and pregnancy rate are expressed by (%). Pairwise comparison of measurement data between groups was analyzed by *t* test. Data were counted by χ^2^ test. Cox regression model includes variables that have influence on recurrence and clinically valuable factors by single factor analysis, and carries out multivariate analysis. The test level was α = 0.05, and *P* < 0.05 indicated that the difference was statistically significant.

## Results

### Comparison of Hormone Levels Between the Two Groups

**Figure 1:**
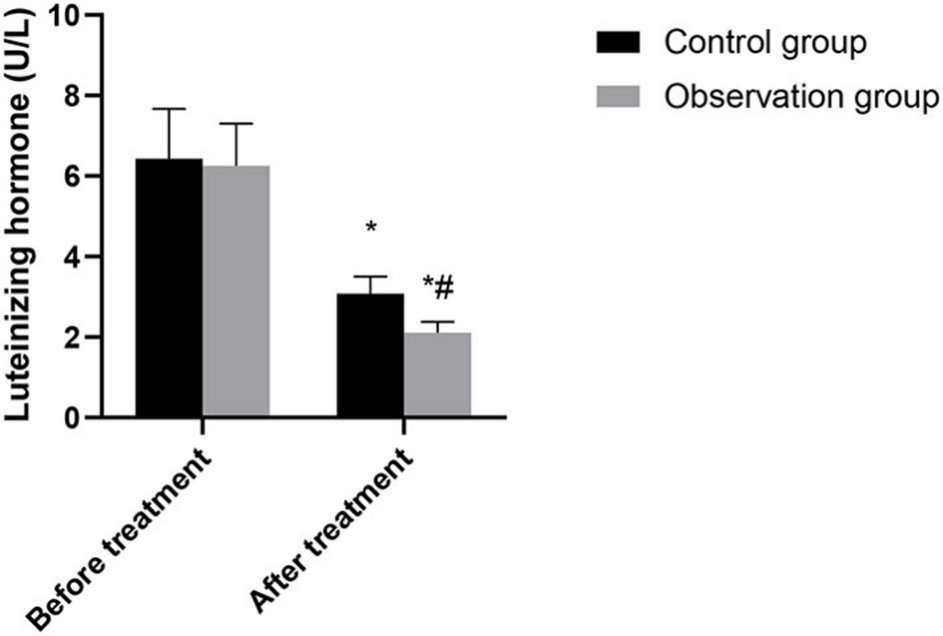
Comparison of luteinizing hormone between the two groups. Compared with before treatment, ^*^P < 0.05. Compared with control group, ^#^*P* < 0.05.

**Figure 2:**
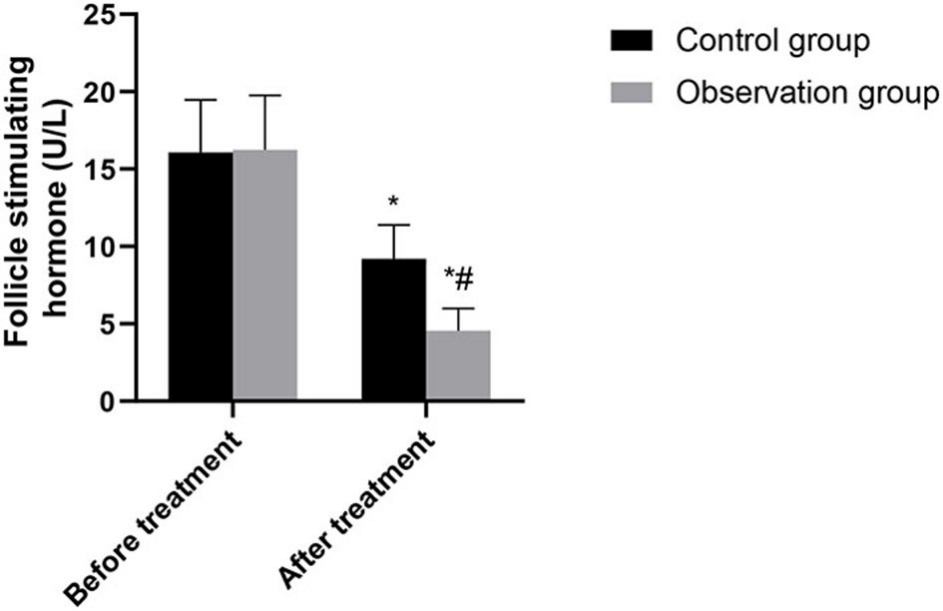
Comparison of follicle-stimulating hormone between the two groups. Compared with before treatment, ^*^P < 0.05. Compared with control group, ^#^*P* < 0.05.

**Figure 3:**
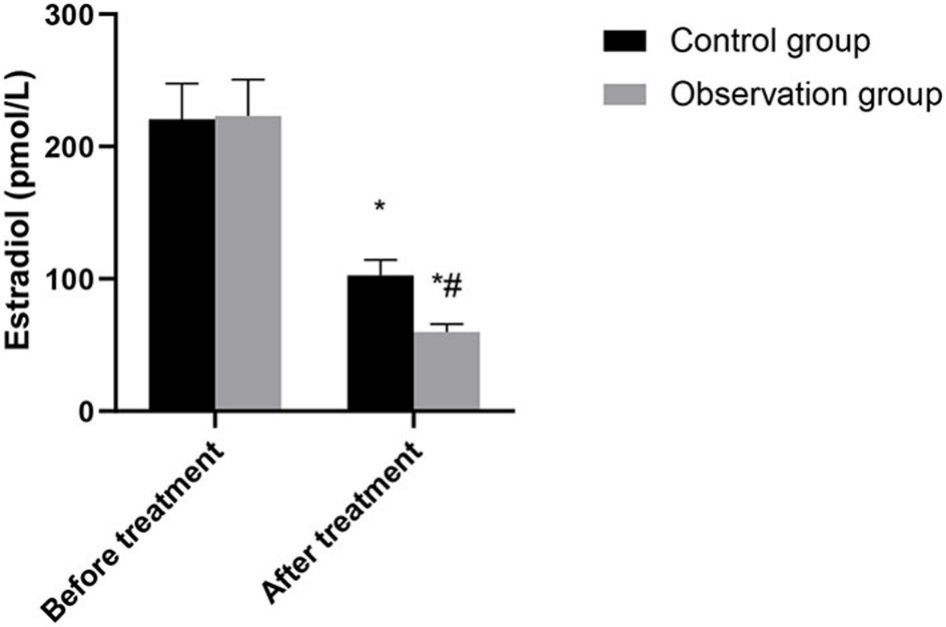
Comparison of estradiol between the two groups. Compared with before treatment, ^*^P < 0.05. Compared with control group, ^#^*P* < 0.05.

There was no significant difference in hormone levels between the two groups before treatment (*P* > 0.05). After treatment, the levels of luteinizing hormone, follicle-stimulating hormone and estradiol in the two groups were significantly lower than those before treatment, and the levels in the observation group were significantly lower than those in the control group (*P* < 0.05). As shown in Figures 1–3.

### Comparison of Recurrence After 12 Months Between the Two Groups

**Figure 4:**
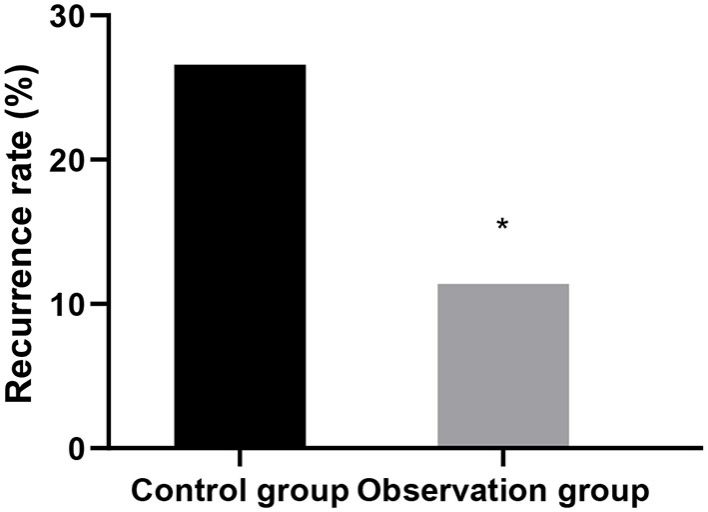
Comparison of recurrence after 12 months between the two groups. Compared with control group, ^*^P < 0.05.

The recurrence rate at 12 months after operation in the observation group was lower than that in the control group (*P* < 0.05). As shown in Figure 4.

### Comparison of the Pregnancy Rates of the Two Groups Within 12 Months After Surgery

**Figure 5:**
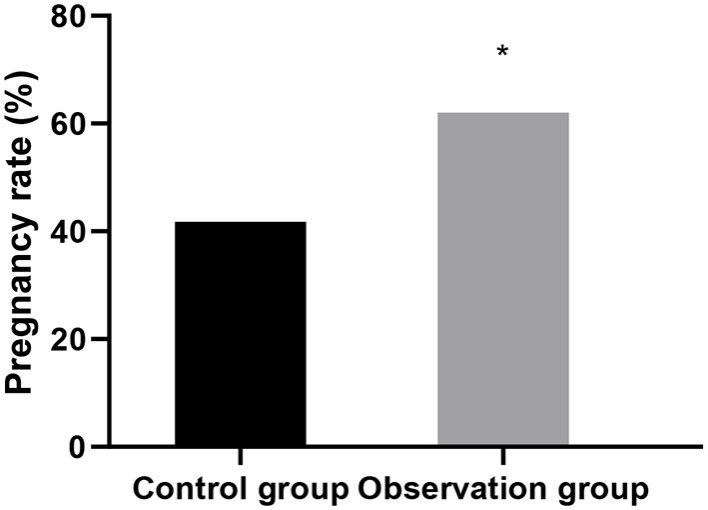
Comparison of the pregnancy rates of the two groups within 12 months after surgery. Compared with control group, ^*^P < 0.05.

The pregnancy rate in the observation group 12 months after surgery was higher than that in the control group (*P* < 0.05). See Figure 5.

### Comparison of the Incidence of Adverse Reactions Between the Two Groups

**Figure 6:**
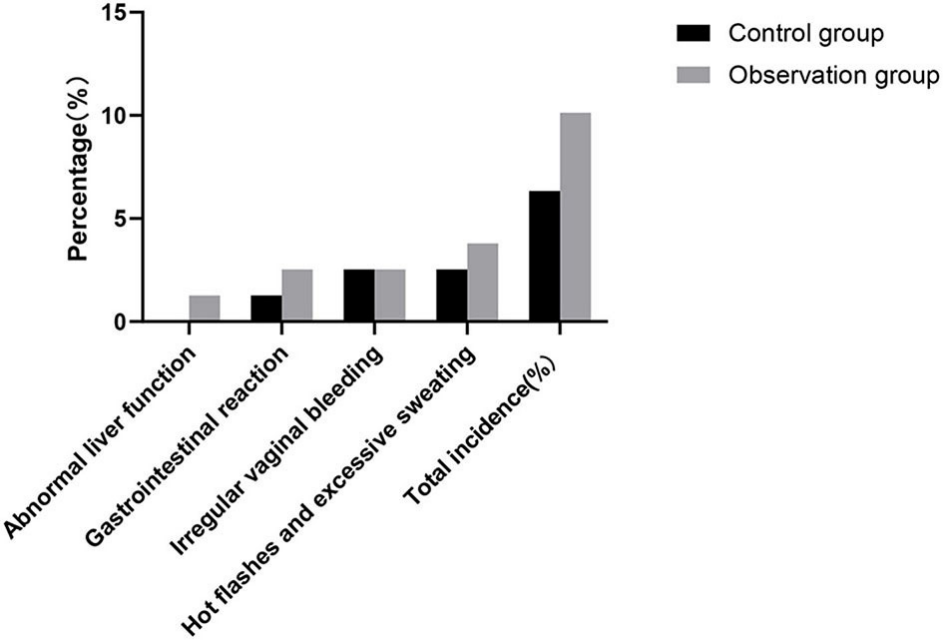
Comparison of the incidence of adverse reactions between the two groups.

The incidence rates of adverse reactions in the control group and the observation group were 6.33 and 10.13%, respectively. There was no significant difference in the incidence of adverse reactions between the two groups (*P* > 0.05). As shown in Figure 6.

### Analysis of Single Factor Affecting Postoperative Recurrence in Patients With Endometriosis

Univariate analysis showed that preoperative dysmenorrhea, postoperative medication, and postoperative pregnancy had an impact on the postoperative recurrence of endometriosis (*P* > 0.05). As shown in Table 1.

**Table 1:** Univariate analysis of postoperative recurrence in patients with ems (*n*,%).

**Factor**	**Recurrence group (*n* = 30)**	**Non-recurrent group (*n* = 128)**	**χ^2^ value**	***P*-value**
Age (years)			2.294	0.130
≥30	17 (56.67)	53 (41.41)		
<30	13 (43.33)	75 (58.59)		
Body mass (kg/m^2^)			2.557	0.110
≤ 24	24 (80.00)	83 (64.84)		
>24	6 (20.00)	45 (35.16)		
Preoperative dysmenorrhea			14.948	<0.001
Yes	26 (86.67)	61 (47.66)		
No	4 (13.33)	67 (52.34)		
r-AFS staging			3.738	0.053
Stage III	11 (36.67)	72 (56.25)		
Stage VI	19 (63.33)	56 (43.75)		
Cyst location			0.336	0.562
Unilateral	18 (60.00)	84 (65.62)		
Bilateral	12 (40.00)	44 (34.38)		
Maximum diameter of cyst (cm)			1.609	0.205
≤ 5	10 (33.33)	59 (46.09)		
>5	20 (66.67)	69 (53.91)		
Cyst rupture			0.834	0.361
Yes	2 (6.67)	4 (3.13)		
No	28 (93.33)	124 (96.88)		
Postoperative medication			5.925	0.015
Yes	9 (30.00)	70 (54.69)		
No	21 (70.00)	58 (45.31)		
Postoperative pregnancy			12.104	0.001
Yes	7 (23.33)	75 (58.59)		
No	23 (76.67)	53 (41.41)		

### Analysis of Multiple Factors Affecting Postoperative Recurrence in Patients With Endometriosis

Multivariate regression analysis showed that preoperative dysmenorrhea was an independent risk factor for postoperative recurrence in patients with endometriosis (*P* < 0.05). Postoperative pregnancy was a protective factor for postoperative recurrence in patients with endometriosis (*P* < 0.05). As shown in Tables 2, 3.

**Table 2:** Assignment for multivariate logistic regression analysis.

**Factors**	**Variables**	**Assignment**
Preoperative dysmenorrhea	X1	No = 0, yes = 1
Postoperative medication	X2	No = 0, yes = 1
Postoperative pregnancy	X3	No = 0, yes = 1

**Table 3:** Multi-factor analysis of postoperative recurrence in patients with endometriosis.

**Variables**	***B***	***S.E***	***Wals***	***P***	***OR***	**95% *CI***
Preoperative dysmenorrhea	−1.284	0.427	9.042	0.015	0.277	0.120–0.639
Postoperative medication	0.841	0.537	2.453	0.241	2.319	0.809–6.642
Postoperative pregnancy	1.314	0.382	11.832	0.008	3.721	1.759–7.867

## Discussion

Endometriosis often occurs in middle-aged women in the growing period. It does not occur before adolescence, while the ectopic focus after menopause shrinks and degrades gradually due to the decline of hormone levels. And the incidence rate is greatly reduced. Although endometriosis is a benign disease, its strong growth and metastasize ability accelerates the development of the disease. Therefore, once the diagnosis is made clinically, effective treatment should be given as soon as possible, and surgical resection is the main treatment (1, 8, 9). Based on the pathogenesis of endometriosis and the treatment principle of laparoscopic surgery, we can know that laparoscopic surgery can loosen the adhered pelvic tissue, clear away the lesions, improve the pelvic environment and promote the recovery of the patient's body, but it can not effectively solve the infertility problem of patients.

In this study, dydrogesterone selected by the control group is a commonly used drug for the treatment of endometriosis. By inhibiting the activity of endometrial and ectopic focus cells, the diseased tissue gradually shrinks, but it can inhibit ovulation and endometrial development, which is not conducive to the implantation of pregnant eggs. At present, gonadotropin-releasing hormone agonists commonly used in clinic include goserelin, triptorelin and leuprorelin. It has been believed by Li et al. (10) that leuprorelin has a relatively mild effect on the ovary, and the incidence of adverse reactions is lower than that of triproline. Leuprorelin belongs to a luteinizing hormone releasing hormone derivative, can treat endometriosis by improving the pituitary function of patient, enhancing the resistance to proteolytic enzyme, reducing the response of women's ovaries, and relieve menstrual disorder caused by ovarian hormone secretion disorder. It is a relatively safe and reliable adjuvant drug for laparoscopic surgery, which can promote the clinical treatment of endometriosis complicated with sterility (11–13).

This study showed that the levels of luteinizing hormone, follicle-stimulating hormone and estradiol in the observation group were lower than those in the control group after treatment. Accurately verified the mechanism of action of leuprorelin, that is, blocking the pituitary-gonadal axis and effectively inhibiting the release of sex hormones, so as to achieve the conditions of feedback inhibition of endometriosis (14). In addition, leuprorelin's treatment of endometriosis can effectively reduce the concentration of endogenous estrogen in patients, reduce the synthesis of protein, lead to atrophy of related tissues, inactivation of nucleic acids and uterine contraction, thus alleviating the symptoms of dysmenorrhea, obesity, infertility andso on (15). In this study, the recurrence rate and pregnancy rate after operation in the observation group were superior to those in the control group. It was confirmed that laparoscopic surgery combined with subcutaneous injection of leuprorelin could significantly improve the infertility and fertility status of patients. There was no significant difference between the two groups in the incidence of adverse reactions. The results showed that Mingliang Bingruilin was safe. After subcutaneous or intramuscular injection, leuprorelin can be hydrolyzed into four degradation products in the body, which are metabolized by the kidney. Therefore, the absorption rate of drug effect is high, and the effect on other organs and tissues of the body is weak, so drug safety is good (16).

At present, there are many versions of the mechanism of endometriosis, among which the widely accepted theory is endometrial implantation, which means that the endometrium debris dropped off during menstrual period flows backwards with the menstrual blood, enters the abdominal cavity through the fallopian tube, and is implanted on the surface of the ovary or other parts of the pelvis (17). After treatment, with the recovery of menstruation, endometrial cells in the menstrual blood may grow to form new endometriosis lesions, and ovulation may also lead to endometriosis. The problem of reducing the recurrence rate of endometriosis after operation has not yet been solved. Therefore, it is particularly important to explore the factors that affect the recurrence of endometriosis after operation, which is helpful to predicting the recurrence risk of patients, and then provide personalized treatment and long-term management for patients. This study showed that preoperative dysmenorrhea was an independent risk factor for postoperative recurrence, and postoperative pregnancy was a protective factor for postoperative recurrence. This result is the same as that reported in previous study (18). Progressive aggravation of dysmenorrhea is a common clinical symptom of endometriosis patients. Inflammatory response plays an important role in endometriosis. Interleukin-1 promotes prostaglandin synthesis leading to dysmenorrhea, while interleukin-6 accelerates the proliferation of ectopic endometrium cells leading to disease progression. Preoperative dysmenorrhea patients may have a more pelvic inflammatory environment, which will promote the emergence of new lesions and the recurrence of residual lesions after operation. As pointed out in the research by Chon et al. (19), dysmenorrhea and peeling of ovarian cyst are closely related to the recurrence rate after surgery. After 6–12 months of surgical treatment, the pelvic environment and ovarian function were improved, which is the key period of pregnancy. Therefore, for patients with endometriosis who are eager to give birth, we should encourage active pregnancy preparation after operation, which can not only effectively prevent postoperative recurrence, but also promote the patients' natural conception.

To sum up, laparoscopic surgery combined with leuprorelin in the treatment of endometriosis complicated with infertility can improve hormone levels of patients, increase pregnancy rate, and have a low recurrence rate. Preoperative dysmenorrhea is an independent risk factor for postoperative recurrence, which should be quantified and included in the recurrence risk prediction model. Postoperative pregnancy can reduce the recurrence rate after surgery, and patients with fertility requirements should be encouraged to actively prepare for pregnancy after surgery.

## Data Availability Statement

The original contributions presented in the study are included in the article/supplementary material, further inquiries can be directed to the first author.

## Ethics Statement

The studies involving human participants were reviewed and approved by the Ethics Committee of the Zhoushan Women and Children Hospital. The patients/participants provided their written informed consent to participate in this study.

## Author Contributions

LY is mainly responsible for the writing of the article and research design. YS is mainly responsible for data analysis. QF is responsible for the guidance of the entire research. All authors contributed to the article and approved the submitted version.

## Conflict of Interest

The authors declare that the research was conducted in the absence of any commercial or financial relationships that could be construed as a potential conflict of interest.

## Publisher's Note

All claims expressed in this article are solely those of the authors and do not necessarily represent those of their affiliated organizations, or those of the publisher, the editors and the reviewers. Any product that may be evaluated in this article, or claim that may be made by its manufacturer, is not guaranteed or endorsed by the publisher.
